# Utilization of an Animated Electronic Health Video to Increase Knowledge of Post- and Pre-Exposure Prophylaxis for HIV Among African American Women: Nationwide Cross-Sectional Survey

**DOI:** 10.2196/formative.9995

**Published:** 2019-05-29

**Authors:** Keosha T Bond, S Raquel Ramos

**Affiliations:** 1 Department of Public Health New York Medical College Valhalla, NY United States; 2 Rory Meyers College of Nursing New York University New York, NY United States

**Keywords:** eHealth interventions, heterosexual, African American women, HIV risk behaviors, HIV prevention, entertainment-education, postexposure prophylaxis, pre-exposure prophylaxis, internet, videos

## Abstract

**Background:**

Despite renewed focus on biomedical prevention strategies since the publication of several clinical trials highlighting the efficacy of pre-exposure prophylaxis (PrEP), knowledge of postexposure prophylaxis (PEP) and PrEP continues to remain scarce among women, especially among African American women who are disproportionally affected by HIV. In an effort to address this barrier and encourage uptake of PEP and PrEP, an electronic health (eHealth) video was created using an entertainment-education format.

**Objective:**

The study aimed to explore the feasibility, acceptability, and preference of an avatar-led, eHealth video, PEP and PrEP for Women, to increase awareness and knowledge of PEP and PrEP for HIV in a sample of African American women.

**Methods:**

A cross-sectional, Web-based study was conducted with 116 African American women aged 18 to 61 years to measure participants’ perceived acceptability of the video on a 5-point scale: poor, fair, good, very good, and excellent. Backward stepwise regression was used to the find the outcome variable of a higher rating of the PEP and PrEP for Women video. Thematic analysis was conducted to explore the reasons for recommending the video to others after watching the eHealth video.

**Results:**

Overall, 89% of the participants rated the video as good or higher. A higher rating of the educational video was significantly predicted by: no current use of drugs/alcohol (beta=−.814; *P*=.004), not having unprotected sex in the last 3 months (beta=−.488; *P*=.03), higher income (beta=.149; *P*=.03), lower level of education (beta=−.267; *P*=.005), and lower exposure to sexual assault since the age of 18 years (beta=−.313; *P*=.004). After watching the eHealth video, reasons for recommending the video included the video being educational, entertaining, and suitable for women.

**Conclusions:**

Utilization of an avatar-led eHealth video fostered education about PEP and PrEP among African American women who have experienced insufficient outreach for biomedical HIV strategies. This approach can be leveraged to increase awareness and usage among African American women.

## Introduction

African American women are disproportionately affected by HIV in the United States, with 91% of new infections among this population being attributed to heterosexual transmission [1]. African American women account for 60% of all new HIV diagnoses among women, yet they do not engage in more sexual risk behaviors than their white and Latina counterparts [2]. Increased risk of HIV among African Americans is because of the high HIV prevalence within their sexual network and other structural factors, such as poor access to health care [3].

For sexually active individuals, the use of the male condom has remained the most cost-effective and readily accessible prevention tool since the start of the AIDS epidemic [4]. The use of condoms among African American women is exacerbated by the complex intersection of gender roles and power differentials between women and men, which may limit women’s ability or willingness to negotiate male condom use or remain abstinent [5]. The inconsistency of condom use among African American women has been linked to their lack of control over sexual health decisions in relationships [3,6]. An option for addressing HIV risk among African American women is the initiation of biomedical prevention strategies such as postexposure prophylaxis (PEP) and pre-exposure prophylaxis (PrEP) for HIV [7]. PEP prevents HIV infection in those who have been exposed by an infected partner within a 72-hour time frame [8]. PrEP consists of taking 1 pill daily to prevent HIV infection before any HIV exposure [8]. Despite FDA approval of PEP in 2005 and PrEP in 2012 [9], both biomedical prevention strategies have been scarcely used among African American women [10].

Little is known about the interest of biomedical prevention strategies among African American women [3,11], suggesting a need for innovative strategies to increase the knowledge and access among women in diverse settings [10,12]. The literature on the acceptability of oral antiretroviral medications as an HIV prevention method among HIV negative, ethnoracial minorities and heterosexual female community members in the United States is scarce [3]. The need to disseminate this information and expand awareness of female-controlled strategies for HIV prevention is limited by the shortage of implementation strategies that target the sexual health goals of heterosexual African American women [3,13-15]. An alternative to traditional methods of health education for expanding awareness and potentially impacting the uptake of these 2 HIV prevention options involves the diffusion of electronic health (eHealth) strategies that is tailored for a particular category of consumers [16].

Recent advances in eHealth have enabled the development of innovative HIV prevention strategies that have created a large number of opportunities for connecting to others to improve health and awareness about HIV prevention [17-19]. eHealth has strengthened in the field of HIV research as more interventions are adopting these techniques to address the barriers to participation in research that addresses sensitive topics [20-22]. Published reports suggested that individuals are more comfortable using computers while addressing sensitive topics such as HIV [23]. Several computer-based interventions have demonstrated efficacy in reducing HIV risk behaviors among African American women [24-26]. Lightfoot et al conducted a 3-arm trial (computer intervention, face-to-face group, and control) of a computer-delivered intervention for a diverse population of predominately African American and Latin youth aged 14 to 18 years [24]. At the 3-month follow-up, students who received the computer intervention were significantly less likely to engage in sexual activity compared with those in the small-group condition and had fewer sexual partners than those in the control condition [24].

Grimley and Hook tested a computer-delivered intervention with primarily African American females who were sexually transmitted infection (STI) clinic patients [27]. At the 6-month follow-up, the study found more consistent condom use and lower STI incidence, 32% for participants in the computer-delivered intervention versus 23% for the participants in the control arm [27]. A randomized controlled trial of Sisters Accessing HIV/AIDS Resources At-a-click (SAHARA), a 2-hour computer-delivered adaptation of the Centers for Disease Control and Prevention (CDC)-Diffusion of Effective Behavioral Intervention (DEBI) evidence-based Sisters Informing Sisters on Topics about AIDS program, found that women in the intervention arm (computer-delivered intervention plus a 20-min small-group discussion) reported a significantly higher percentage of condom-protected sex acts than those in the control arm [26].

Sisters Informing Healing Living and Empowering (SiHLE), a 4-session CDC-DEBI group-level HIV prevention intervention for teenage African American females aged 14 to 18 years, was translated into a 2-hour computer-delivered individual intervention called multimedia SiHLE [25]. The results of this study showed that the average condom-protected sex acts (proportion of vaginal sex acts with condoms in the last 90 days) for sexually active participants receiving multimedia SiHLE increased from 51% at baseline to 71% at 3-month follow-up, yet no statistically significant difference was found in the control group [25]. Nonsexually active intervention group participants reported a significant increase in condom self-efficacy, and there was no statistically significant difference found in the control group [25]. The study provided preliminary support for the efficacy of a computer-delivered adaptation of a proven HIV prevention program for African American teenage women [25]. This is consistent with meta-analyses that have shown that computer-delivered interventions, which can often be disseminated at lower per-capita cost than human-delivered interventions, can influence HIV risk behaviors in a positive fashion [28]. These studies suggested that computer-delivered interventions have the potential to be at least as effective as human-delivered interventions in influencing HIV risk behavior [22,28]. Unfortunately, African Americans and other marginalized racial/ethnic populations have not fully benefited from eHealth interventions [29].

Despite the fact that computer-based interventions have been efficacious among African American women [22], current Web-based HIV prevention research has been with predominantly white participants [30]. It has been suggested that African Americans have not been represented in Web-based interventions because of disparities in internet access owing to lower socioeconomic status [31]; yet, research has shown that there is no distinguishable difference in social media use among ethnicities [32]. This may be due to the limited data on understanding their use of technology and willingness to participate in eHealth/mobile health research [33]. With African Americans’ increasing use of the internet through mobile technology and African American women’s frequent use of the internet to search for health information, there is an opportunity to engage African American women in research through an eHealth intervention for HIV prevention while minimizing some of the common barriers to community-based and clinical interventions [1,33]. To directly address the quandary of little to no awareness of biomedical prevention strategies for HIV prevention among African American women, our study sought to strengthen knowledge of PEP and PrEP using eHealth technology in an internet-based research study. Thus, the purpose of this study was to assess the feasibility and acceptability of the *PEP and PrEP for Women* video, which was designed to increase the knowledge of PEP and PrEP using entertainment-education format eHealth targeting African American women.

This study was guided by the social cognitive theory through the use of the entertainment-education communication strategy [34-36]. The social cognitive theory posits that individuals learn through the observation of others’ attitudes, behaviors, and the outcomes of those behaviors [34]. In other words, behaviors are learned either by modeling the behavior of others or by direct experience [34]. Entertainment-education is a theory-based communication strategy that embeds educational and social narrative messages into a popular entertainment format such as media to achieve desired individual, community, institutional, and societal changes among the intended media user populations [36]. Previous research has shown that observing others enact a health behavior via entertainment-education narratives can significantly increase the viewer’s health knowledge, attitudes, intentions, and behavior [37].

Entertainment-education narratives exert influence by reducing the audience’s resistance to health messages [36]. The central constructs of the social cognitive theory are self-efficacy, the belief in the ability to implement the necessary behavior, and outcome expectancies [34]. It has been found that a strong sense of personal efficacy is related to better health, higher achievement, and more social integration [38]. The learning process is facilitated through combining storytelling and reflection [39]. This represents an active, purposive, contemplative, and deliberative approach where meaning is generated by the learning experience [39]. Entertainment-education through digital storytelling engages learners; organizes information; facilitates remembering; enhances discussion, problem posing, and problem solving; and aids understanding of the content [36,40].

In our application of the social cognitive theory framework and entertainment-education narratives, the *PEP and PrEP for Women* eHealth video study is intended to engage learners through digital storytelling and critical thinking on issues about (1) HIV risk, (2) sexual violence, (3) relationship dynamics, (4) patient-provider communications, (5) initiation of PEP and PrEP, and (6) female-controlled HIV prevention methods. A collaborative process among the research team members resulted in an initial 9-min and 3-second script that was based on a review of the HIV risk literature on African American women and their male sexual partners [5,15,41].

## Methods

### Recruitment

This study utilized data collected over a 6-week period (February and March 2015) for a cross-sectional health survey conducted on the Web. Participants were recruited through a social marketing campaign, which included Web recruitment via social media, flyers posted in community settings, and snowball sampling. Web-based venues used in Web recruitment included Facebook, Twitter, LinkedIn, and Craigslist. Flyers were posted in community venues that African American women frequent (eg, churches, hair salon, health centers, and colleges in the New York metropolitan area). As this is a historically difficult-to-reach population, snowball sampling was used to recruit African American women [42]. Study eligibility was as follows: (1) reported female identity, (2) self-identifying as black or African American, (3) aged 18 years or older, (4) ability to read English, and (5) willingness to participate. Those who clicked on the recruitment banner were routed to a screening survey on a secure study website. A total of 384 individuals consented to participate in the study. Of those, 34.3% (132/384) individuals were ineligible after not meeting the inclusion criteria. An additional 10.9% (42/384) of the surveys had missing data and, therefore, were also ineligible to be included in the sample. Furthermore, 7 eligible surveys were identified as duplicate responses based on identical IP addresses and were excluded from the sample (n=116). Moreover, per our original inclusion criteria, participants who did not watch the entire video were excluded from the regression model to ensure that the participants in the analysis received the same exposure during the study. As such, the final sample size was N=91.

Those who were deemed ineligible were thanked for their time and exited the site. Eligible participants were directed to the Web-based consent form, which outlined the study’s purpose, risks, and benefits followed by a prevideo survey, a 9-min eHealth video (PEP and PrEP for Women), and a brief postvideo survey. Those who completed the study were entered into a random drawing to receive an Amazon gift card (US $100, US $200, or US $300) and asked to forward the link to potential eligible participants in their network. The institutional review board at Teachers College Columbia University reviewed and approved all study procedures.

### Prevideo Survey Assessment

We assessed a comprehensive range of sociodemographic variables, including age, employment status (unemployed vs employed), annual income level (from “no income” to “more than $50,000” in increments of $20,000), education level (high school/GED or less vs some college vs college or more), current student (yes/no), relationship status (single vs partnered), and gender of current sexual partners (male, female, or both). We also assessed previous knowledge of both PEP and PrEP. Additional information related to sexual risk behaviors including drug/alcohol use, drug/alcohol use during sex, HIV testing history, relationship dynamics [43], condom use, self-efficacy for condom use [44], history of adult and child sexual abuse, and sexual assault within their social network was collected in the prevideo survey and entered into the regression model but not reported in this paper. After the completion of the prevideo survey, participants were then directed to the 9-min *PEP and PrEP for Women* eHealth video.

### Electronic Health Video

The 2-part *PEP and PrEP for Women* eHealth video was created using Goanimate Technology [45]. Goanimate is a software program used to create professional, animated, avatar-led videos with an entertainment-education style [45]. Research supports the premise of technology-based approaches [45] to facilitate learning and decision making.

Part 1 of the video addresses a storyline that warrants the initiation and use of PEP. In scene 1, the main character, Tania, informs her friend, Michelle, that she has been sexually assaulted (Multimedia Appendix 1). In scene 2, Tania is in the emergency room speaking with her physician after being examined. The physician explains how PEP could be used by women who may have been exposed to HIV. Tania decides to begin PEP treatment to prevent HIV from her possible exposure. In scene 3, Tania returns for a follow-up visit 28 days after she started her PEP regimen at the hospital clinic. This concluded part 1 of the eHealth video.

Part 2 addressed a storyline that warrants the use of PrEP to prevent HIV transmission. In scene 4, we introduce 2 new characters for this narrative, Kia and Jackie. The main character Kia has a discussion with her friend, Jackie, about her boyfriend Mike (Multimedia Appendix 2). Mike has not been monogamous in his relationship with Kia, and she is concerned. Jackie informs Kia about PrEP and suggests that she talks to a health care provider about initiating PrEP to protect her from possibly getting HIV. In scene 5, Kia’s physician explains the history of PrEP and how PrEP can be used by women who are in a relationship with an HIV-positive partner or a nonmonogamous partner who may be at high risk of obtaining and transmitting HIV, such as in Kia’s current situation. In addition, Kia’s physician discusses some common concerns about using PrEP, such as cost, side effects, and the routine laboratory tests that are required as a part of treatment. Finally, Kia’s physician empowers her with this information and lets her decide what is best for her—either starting prophylactic treatment now or considering its future use. This concluded part 2 of the eHealth video.

### Postvideo Survey

After the participants watched the video, we conducted a brief survey to measure the participant’s perceived acceptability of the video on a 5-point scale. First, we accessed their dose of exposure to the video to identify if the participants watch all, most, some, or none of the video. Second, we asked participants to rate the video with the following response anchors: poor, fair, good, very good, or excellent. Next, participants were asked if they would recommend the video to other women. A dialogue box was provided to ascertain why or why not they would share this video and information with other women. Finally, they were asked about their intentions to use PrEP and PEP in the future, if needed, and if they would recommend PrEP and PEP to other women.

### Quantitative Data Analysis

Quantitative data were downloaded from SurveyMonkey, transferred to SPSS, and analyzed using SPSS 21 [46]. Descriptive statistics, including frequencies and proportions, were computed for all sociodemographic variables. Tabulations were performed for all responses. Bivariate correlations were calculated among the study variables using the Pearson coefficient of correlation (Pearson r). The outcome variable of interest was the participant’s ratings of the *PEP and PrEP for Women* eHealth video. To refine our model to exclude insignificant or highly correlated variables, backward stepwise regression was used. The rationale for using this approach was based on the work by Mantel who explained that backward selection serves to reduce the degrees of freedom, has joint predictor capability, and removes noise caused by including unrelated variables or variables that may be highly correlated with each other [47]. This was conducted by including the full group of 22 predictor variables that were all entered into the equation regardless of their worth for the creation of the regression model. The backward stepwise regression analyses included 22 independent variables as potential predictors: (1) age, (2) level of education, (3) annual household income, (4) has a current partner, (5) is a student, (6) employed, (7) relationship status, (8) lifetime HIV testing, (9) tested positive for an STI, (10) current drug use, (11) have other sex partners, (12) had any unprotected sex with main/steady and any other partner(s) in the past 3 months, (13) heard of PEP before watching the video, (14) heard of PrEP before watching the video or not, (15) higher percentage of time had sex while using alcohol/drugs, (16) prevalence of exposure to sexual assault/rape before 18 years old, (17) prevalence of exposure to sexual assault/rape since 18 years old, (18) prevalence of sexual assault experiences, (19) prevalence of sexual assault in their social network, (20) self-efficacy for condom use [44], (21) better partner characteristics [43], and (22) more self-control in a relationship [43]. Next, the backward stepwise method involved the least significant variable (one with the largest *P* value) being removed when the model was refitted. Then, a new model is built in the absence of that 1 independent variable and the evaluation process is repeated again, removing the least significant variable. This removal process and the equation-reconstruction process were continued until only significant independent variables (*P*<.05) remained—as the final model reported for the backward stepwise regression.

### Qualitative Data Analysis

Analyses of the open-ended questions were thematic, focusing on dominant themes that emerged and were organized by the question asked; attention was paid to the level of endorsement of a theme across the sample. For the qualitative analysis, a grounded theoretical approach was used, permitting the use of categorizing strategies to code and analyze the data [48,49]. First, the transcripts were summarized in a *digest* that identified the major themes of the responses. Using QSR International’s NVivo 9 qualitative software, the coding of the data was conducted in 3 steps. First, based on the transcripts of the responses, a list of analytic areas represented in the data was composed and given a code (ie, a *closed code*) derived from relevant literature pertaining to entertainment-education narratives [36]. Second, the primary analyst (principal investigator) reread the transcripts and identified blocks of text to be given a descriptive label (ie, either a label from the closed code list or an original one, termed an *open* *code*). Next, the open-coded data were organized under and integrated into the closed code list. Third, the data under each thematic code (eg, *empowering*, *educational* / *informative*, *usability*, *suitability*, and *entertainment*) were reread, and, if needed, recoded into subcategories to refine the analytic categories used. As the themes of the responses emerged, special attention was paid to data that did not confirm emerging themes, noting these for exploration in future research. In addition to using these methods of analysis, we also ensured analytic rigor by engaging several peer reviews of early analytic claims [50]. For this analysis, we will focus on the questions related to recommendations for use of the video.

## Results

### Characteristics of Overall Sample

In our sample (N=116), the mean age was 34 years. Most women were born in the United States (109/116, 94%), identified as black or African American (115, 99%), were in a relationship (71, 61%), and were intimate with male partners only (110, 95%). Approximately one-third (30, 27%) of them had a high school education, and a similar proportion (38, 33%) of them were current students. The majority (83, 72%) of them reported either current full-time or part-time employment. Almost half (52, 47%) of them reported a household income of US $50,000 or more. Before watching the video, only 18% (21) of the participants reported that they heard of PrEP and 22% (26) reported that they heard of PEP (Table 1).

**Table 1:** Demographic characteristics of a Web-based sample of African American women.

Characteristic		Watched entire video			*P* value
		Total (N=116)	Yes (n=91)	No (n=25)	
Age (years), mean (SD)		34 (10.3)	34 (10.5)	33 (9.6)	.70
**US born, n (%)**					
	Yes	109 (94.0)	84 (92)	25 (100)	.15
**Education (n=111^a^), n (%)**					**.15**
	High school or less	30 (27.0)	21 (23.9)	9 (39.1)	
	Trade school/associates	17 (15.3)	16 (18.2)	1 (4.3)	
	College degree or more	64 (57.7)	51 (58.0)	13 (56.5)	
**Income (n=112^a^), n (%)**					**.20**
	≤US $19,999	21 (18.8)	16 (18.0)	5 (21.7)	
	US $20,000-US $39,999	29 (25.9)	27 (30.3)	2 (8.7)	
	US $40,000-US $49,999	10 (8.9)	7 (7.9)	3 (13.0)	
	US $50,000 or more	52 (46.4)	39 (43.8)	13 (56.5)	
**Employment, n (%)**					
	Employed	83 (71.6)	63 (69.2)	20 (80.0)	.29
**Student, n (%)**					
	Current	38 (32.8)	34 (37.4)	4 (16.0)	.04
**Relationship status, n (%)**					**.21**
	Single	45 (39.0)	38 (41.8)	7 (28.0)	
	Partnered	71 (61.2)	53 (58.2)	18 (72.0)	
**Sex partners’ gender, n (%)**					**.19**
	Male	110 (94.8)	85 (93.4)	25 (100.0)	
	Both male and female	6 (5.2)	6 (6.6)	0	
**PEP^b^ and PrEP^c^, n (%)**					
	Previous knowledge of PEP	26 (22.4)	19 (20.9)	7 (28.0)	.45
	Previous knowledge of PrEP	21 (18.1)	17 (18.7)	4 (16.0)	.76

^a^The n value is different because of missing data.

^b^PEP: postexposure prophylaxis.

^c^PrEP: pre-exposure prophylaxis.

### Dose of Exposure to Electronic Health Video

Overall, 78% (91/116) of the eligible participants watched all of the video (Table 2). In addition, 11% (13) of them watched most of the video and 3% (3) watched some of the video. Furthermore, 8% (9) of the eligible participants did not watch any of the video (Table 2). Analyses of the postvideo survey and regression model only included those who reported watching the entire eHealth video.

**Table 2:** Dose of exposure to the video (N=116).

Dose of exposure	n (%)
I watched all of the video	91 (78.4)
I watched most of the video	13 (11.2)
I watched some of the video	3 (2.6)
I watched none of the video	9 (7.8)

### Rating and Recommendation of Electronic Health Video

Overall, 89% (81/91) of the participants rated the video as good or higher; 29% (26) of them rated the video as excellent, 32% (29) rated the video as very good, and 29% (26) rated the video as good. Only 10% (9) of participants rated the video as fair and 1% (1) rated the video as poor. The mean rating for the video was very good (mean 4.77, SD 1.01). Among the sample, 91% (83) of participants reported that they would recommend the video to others, whereas 9% (8) of them reported that they would not recommend the video.

After watching the video, 97% (88/91) of participants reported that they would seek out PEP if they felt that they might have been exposed to HIV, including taking the recommended oral medication for 28 days. In addition, 93% (85) of them reported that they would recommend to another woman to seek out PEP if they knew the woman might have been exposed to HIV. Regarding the use of PrEP, after watching the video, 76% (69) of them reported that they would seek out PrEP. In addition, 91% (83) of participants reported that they would recommend to another woman to seek out PrEP (Table 3).

**Table 3:** Rating of the video and acceptability (N=91).

Characteristics		n (%)
**Video rating^a,b^**		
	Poor	1 (1)
	Fair	9 (10)
	Good	26 (29)
	Very good	29 (32)
	Excellent	26 (29)
**Video acceptability**		
	Yes, I would recommend	83 (91)
	No, I would not recommend	8 (9)
**PEP^c^ and PrEP^d^ acceptability**		
	Would seek out PEP after watching the video	88 (97)
	Would recommend PEP to other women	85 (93)
	Would seek out PrEP after watching the video	69 (76)
	Would recommend PrEP to other women	82 (90)

^a^Mean 4.77, min 2, max 6, SD 1.01.

^b^Percentages do not total 100 due to rounding errors.

^c^PEP: postexposure prophylaxis.

^d^PrEP: pre-exposure prophylaxis.

### Backward Stepwise Regression

Regression analysis identified the best predictors for giving the PEP and PrEP video a higher rating (ie, good, very good, and excellent). The backward stepwise regression analysis showed that the 5 variables that best predicted a high rating of the video were not engaging in any current drug use (beta=−.814; *P*=.004), using condoms all the time in the past 3 months (beta=−.488; *P*=.03), a higher household income (beta=−.149; *P*=.03), lower education level (beta=−.267; *P*=.005), and less experience of sexual abuse as an adult (beta=−.313; *P*=.004). For this model, the adjusted R square was .224 (*F*=6.067; *P*<.001; df=5), meaning that 22.4% of the variance was explained by this regression model (Table 4).

**Table 4:** Backward stepwise regression predicting a higher rating of the PEP (postexposure prophylaxis) and PrEP (pre-exposure prophylaxis) for Women eHealth video.

Variable	Beta	Standard of error	*t* test	*P* value
Drug use	−.814	0.277	−2.939	.004
No condom use	−.488	0.214	−2.277	.03
Income	−.149	0.066	2.267	.03
Education	−.267	0.092	−2.887	.005
Sexual abuse	−.313	0.104	−3.005	.004

### Qualitative Analysis of Video Recommendations

Thematic analysis was used to analyze the open-ended questions to identify reasons for recommending the video to other women. Overall, 91% (83/91) of the participants expressed that they would recommend the video to other women. Our analysis yielded 3 themes about the eHealth video, which were (1) educational, (2) entertaining, and (3) empowering (Table 5).

Under the first theme, educational, subthemes covered how the video provided awareness or knowledge of PEP and PrEP and how it was adaptable. The participants reported that they were not aware of PEP and PrEP before watching the video and that the video was informative and easy to follow. For the second theme, entertaining, subthemes covered how the video had characters that were relatable, and the information was provided via a video with a storyline and characters. The participants reported that they would recommend the video because the *at-risk* scenarios were relatable to women’s current relationship situations, and it may encourage them to change their behaviors. Other participants commented that the video was nice to watch and entertaining. For the third theme, empowering, subthemes covered 3 areas: (a) female-controlled HIV prevention methods, (b) culturally relevant, and (c) heightened perception of risk. The participants reported that they would recommend the video because it provided information on an HIV prevention method that was female controlled. In addition, the video was considered to be culturally relevant based on the scenarios depicted in the video, and it was not judgmental. Finally, the participants reported that the video provides scenarios that would heighten women’s awareness of their own risk because of their partner’s behavior in sexual relationships.

In addition, 8 participants reported that they would not recommend the video to other women (information was excluded from the table). There were 3 emergent themes, including subthemes. First, participants expressed criticism of the script—with the subtheme of their identification with all of the characters. One participant wrote regarding the characters in part 2:

I would prefer more back and forth between women who are considering it, not a doctor promoting it.

Participants stated that they would have preferred for the health information to not be delivered by the doctor character in the video. Second, participants reported problems maintaining attention because of the length of the video:

Although this video is very informative, it is too long. I doubt people will watch the whole thing.

Third, participants expressed a preference for other modes of communication—with subthemes of age appropriateness and video format. The participants reported that the animated format was too cartoonish and would be more appropriate for younger women. As expressed by 1 of the participants:

While it was educational, I feel the avatar format is better suited for middle-older adolescent/college age women. The video was done very well though.

**Table 5:** Reasons for recommending the PEP (postexposure prophylaxis) and PrEP (pre-exposure prophylaxis) for Women eHealth video.

Category, theme, subtheme		Sample quotes
**Education: informative/accessible**		
	Awareness of PEP and PrEP	“As an 18-year-old still in high school and still currently learning about sexual education; I think it’s important for the girls around me and myself to learn about these new drugs (at least new to me) so we can stay informed when engaging in sexual activities.”
	Adaptable	“Good conversation starter. I thought the video was extremely informative. It was easy to follow and can help any woman who may have questions regarding PEP/PrEP.”
**Entertainment: entertaining/engaging**		
	Relatable characters	“They were cute and personable.”
	Format: use of avatars	“Easy to understand and associate with the avatars.”
**Suitability: helpful/empowering**		
	Female-controlled prevention method	“It is important for all women because no man is going to be 100% honest with you if they are sleeping with other individuals.”
	Culturally relevant	“It is important that women are aware of the existence of preventive care for two distinctly different sexual situations…. I like the fact that this is culturally relevant and reflects a nonjudgmental approach.
	Heightened perception of risk	“Good information about PrEP and PEP. Also may inspire women to leave unhealthy relationships after seeing how the PrEP candidate sounded in her justification of staying in her unhealthy relationship.”

## Discussion

### Principal Findings

This intervention aimed to increase the knowledge and initiation of PEP and PrEP among African American women by modeling situations and discussions that would be relevant to the target population. The goal was to also make the video entertaining (ie, *edutainment*) and engaging, while having diverse characters suited for connecting to African American women [51]. More specifically, the intervention included personal narratives that were culturally tailored to the experience of HIV epidemic among African American women and provide information on PEP and PrEP [52]. For example, in *PEP and PrEP for Women*, the viewer learns through a conversation between 2 friends (Kia and Jackie) that the main character, Kia, does not have to rely on her male partner to use condoms to prevent HIV. In this scene, Kia learns about PrEP from her friend, and she is referred to a clinic that provides PrEP. The video does not attempt to answer the question of personal responsibility or criticize complex relationship dynamics that influence HIV risk, but rather it increased the viewer’s knowledge and awareness of an alternative HIV prevention option via involvement with the story characters [39]. Furthermore, the viewers may identify with the protagonists of the stories and thus might be better able to relate the scenario to their own lives while lessening the resistance to the health messages [40].

The participants reported a positive perception of the video, with 89% of those who watched all of the video reporting a video rating of good or higher. In addition to rating the video, 91% of them reported that they would recommend the video to other women. Moreover, 4 salient factors facilitated the recommendation of the video by participants. The high acceptability of the video could be attributed to its focus on providing educational messages in an entertainment content to raise awareness and increase knowledge of 2 novel HIV prevention strategies, PEP and PrEP. First, the information provided on PEP and PrEP increased their knowledge of novel prevention strategies [53]. Second, the format of the video was inclusive of phenotypically relatable characters [54]. Third, the animations and subject matter were entertaining using visual storytelling and the information was valuable [55]. Finally, the subject matter was culturally relevant [52] and focused on a topic about female-controlled HIV prevention methods [15].

Results from regression analysis showed that the major predictors of giving the video a high rating included not having unprotected sex in the past 3 months, lower education, higher income, less sexual abuse as an adult, and no current use of drugs. This could be interpreted that women who would be not considered at high risk for HIV or potential candidates with PEP or PrEP were more receptive to the video. Previous research has shown that people who are already receptive to or substantially aware of the issues were more inclined to adhere to the health message and evaluate it favorably [56].

It is important to acknowledge that African American women are disproportionately affected by HIV not only because of behavior but also because of vulnerabilities created by unequal cultural, social, and economic status [57]. Our sample represents an atypical Web-based sample of African American women with high socioeconomic status, including high levels of education, employment, and income [58]. The higher socioeconomic status is perhaps reflective of attributes of those who have access to computers and the internet or the impact of the snowball data collection method, resulting in a network of women with high incomes participating in the study. However, the high socioeconomic status of the sample may have important implications that are reflected in the body of data obtained. Very few studies have focused on HIV/AIDS risk among African American women with higher socioeconomic statuses and the challenges that they may face in their environment that are similar to women of color with lower socioeconomic statuses [59,60].

The content addressed in the video, information on HIV prevention methods that were female controlled, was provided as one of the reasons that the participants would be willing to recommend the video to other women. Among the sample, there was low awareness of both PEP and PrEP before participating in the study. This mode of eHealth videos that incorporate entertainment-education dramas can persuade individuals because they depict characters who changed their behavior by initiating PEP and PrEP to improve their lives [61]. Drama incorporates a component of emotional response to the informational content, and the combination of emotion and information works together to promote individual intentions to engage in prevention activities [61,62]. The participants reported that the characters were relatable and they had personal knowledge of similar situations experienced by the characters in the PEP and PrEP for Women video. After viewing the video, 88% and 69% of the participants reported that they would seek out PEP and PrEP, respectively, for themselves. This is reflective of the impact of entertainment-education used as a tool to change health behavior [63,64]. Bandura recommended that characteristics of the models should be similar to the viewers to increase the impact of educational modeling [64]. Perhaps the most important lesson is that although a Web-based intervention relies on audiovisual content, it is also important to tailor the intervention to the target population.

A generally positive response to eHealth as an innovation for disseminating HIV prevention was found in this study, but 22% of the participants reported that they did not watch all of the video. There are several possible reasons for why the individuals did not completely watch the eHealth video. Participants who were not able to watch the entire video expressed that they experienced technical problems with the video, which included not being able to watch the video on specific handheld devices. This issue could frustrate participants and cause them to skip over watching the video to complete the survey. An additional reason for not completing the video could be participant’s fatigue because of the length of the video. In this study, some of the anticipated issues related to fatigue were addressed by creating 1 long video instead of 2 different videos for PEP and PrEP, but the length of the video was cited as a reason to not recommend the video. Participants reported that the video was informative, but the length of the video may make it difficult for viewers to maintain their attention. Other reasons cited for not recommending the video included the educator/health care provide character and the use of animation. Participants felt that the PEP and PrEP educator character should have been a peer and not a health care provider. Previous research reported that groups that have traditionally lacked power, such as African American women, may be more sensitive to an interventionist’s physical characteristics [65]. Despite the fact that the doctor in the *PEP and PrEP for Women* video is an African American woman, the participants felt disengaged from the character. This may be reflective of the lack of cultural competence among medical providers, institutional racism, and personally mediated racism and sexism that have negatively affected the sexual and reproductive health of African American women [57]. Participants also stated that the animated format was not appropriate for all age groups. They reported that it was more suitable for teenagers or younger adults.

Although some consumers may criticize the limitations of the technology (eg, age appropriateness), the vast majority found the avatar video product to be informative, accessible, and engaging. Those individuals who have a positive response to the avatar video were willing to adopt the innovation and engage in the diffusion of it within their social networks, which includes disseminating on social media networking sites. Studies have shown that HIV prevention programs grounded in the diffusion of innovation theory can be useful for increasing HIV/AIDS risk reduction knowledge among early adopters and diffusing information throughout social networks; therefore, preventing HIV among vulnerable populations [66]. This study specifically demonstrated how programs may need to strive to incorporate social media technologies to disseminate information. In addition, prevention programs should also take relationship status into consideration during planning and implementation of effective programs to reduce women’s HIV/AIDS risk.

Finally, this study offers recommendations and suggestions for Web-based research as well as Web-based recruitment using modern-day social networking. Although the sample may not be representative of all African American women in the United States with respect to socioeconomic status, this strategy allowed for rapid recruitment of the sample population with minimum resources, such as staff and funding. Furthermore, this strategy potentially provides opportunities not only for recruitment purposes but for eHealth intervention purposes. Researchers and practitioners should consider the benefits of not only Web-based survey research but also of Web-based health education, health promotion, and disease prevention to reach more diverse samples of subpopulations affected by the HIV epidemic. eHealth intervention has proven to be extremely useful in educating African American women on a number of topics related to HIV/AIDS or STI prevention. Web-based health education can be beneficial for African American women if they use the internet to access and share information with their peers. In addition, as previous research studies have shown, using eHealth interventions may help reach a subgroup of African American women who are not usually addressed in the literature pertaining to HIV risk and prevention [67].

### Study Limitations

This study had some limitations. First, this was a small, cross-sectional, Web-based sample of African American women, thus making it difficult to reach generalizations from this data source. Second, internet access familiarity with Web-based technology may have influenced the decision to participate in the study. Women who use a different type of electronic device (eg, cell phones and tablets) to access the internet may be able to navigate websites easily and may encounter challenges with technical issues. Third, the video and script were not previously tested with different groups of African American women. Having focus groups or concept-mapping sessions before the launch of the study would have enhanced the script of the video for tailoring of the message. Future research should involve conducting focus groups with different age groups, socioeconomic statuses with regard to the scripting, video display, and avatar characters to reach a broader audience of African American women. Fourth, given that this is a feasibility and acceptability study, it is not possible to make any conclusion regarding the efficacy of the video. In addition, participants’ responses to PEP and PrEP adoption intentions were based on a hypothetical situation rather than their actual experience. Increasing knowledge does not necessarily correlate with motivating behavioral changes. Finally, the results could have been influenced by social desirability bias [68]. To minimize bias, participants were able to anonymously take the survey on their 1 device. Despite these limitations, the results of this study have important implications for interventions targeting HIV risk behavior among African American women. In addition, it also has important implications for utilizing technology to disseminate HIV prevention information to this population [69].

### Conclusions

This study has demonstrated that the *PEP and PrEP for Women* video using eHealth and entertainment-education health message narratives for a targeted, culturally centered, HIV/AIDS preventative education intervention for African American women can be readily adopted and diffused as an innovation, while still using low-cost technology in the public domain. Findings from this eHealth video study demonstrated that (1) a culturally tailored eHealth video could be used to increase awareness of HIV prevention methods and (2) it is feasible to recruit and engage African American women into an eHealth intervention that is delivered via the internet and social media. The *PEP and PrEP for Women* video was viewed as culturally centered Web-based content by the participants and capitalized on the widespread use of the internet to reach a population that is not only underserved in eHealth interventions but also in traditional HIV research targeting African American women, therefore, adding to the limited body of research on computer-mediated HIV interventions targeting African American women [1,67]. The eHealth video is an acceptable mode of education to address HIV prevention methods. It follows the CDC’s recommendation to create health messages that are relevant, useful, and interesting to encourage the audience to interact and be engaged [52].

Given the disproportionate impact of the HIV epidemic on African American women, the lack of awareness of both PEP and PrEP [3], and poor participation rates of minority populations in prevention interventions [33], it is essential to develop strategies to reach underserved groups and reduce some of the challenges associated with face-to-face preventive interventions. eHealth technologies have the potential to reduce the gap between what is known about PEP and PrEP and its utilization among high-risk populations by increasing individuals’ sexual health knowledge. Future research is warranted on improving the tailoring and design of the eHealth video by including African American women and other underrepresented groups, such as transgender individuals, to participate in the design of an updated PEP and PrEP for HIV video. This can be explored through formative qualitative research to first assess the cultural and structural factors that may influence uptake of both PEP and PrEP.

## Appendix

Multimedia Appendix 1 Screenshot of postexposure prophylaxis (PEP) and pre-exposure prophylaxis (PrEP) for Women eHealth Video Part 1-Post-exposure prophylaxis: Physician talking to Tania about the PEP treatment.

Multimedia Appendix 2 Screenshot of the postexposure prophylaxis (PEP) and pre-exposure prophylaxis (PrEP) for women eHealth Video Part 2-Pre-exposure prophylaxis: Kia and Jackie having a conversation about Kia’s boyfriend’s infidelity.

## Abbreviations

CDC: Centers for Disease Control and Prevention
DEBI: Diffusion of Effective Behavioral Intervention
eHealth: electronic health
PEP: postexposure prophylaxis
PrEP: pre-exposure prophylaxis
SiHLE: Sisters Informing Healing Living and Empowering
STI: sexually transmitted infection
