# Supplementary figures and images for: Utilization of an Animated Electronic Health Video to Increase Knowledge of Post- and Pre-Exposure Prophylaxis for HIV Among African American Women: Nationwide Cross-Sectional Survey
Source: JMIR Form Res. 2019 May 29;3(2):e9995. doi: 10.2196/formative.9995 (PMC6658301; doi:10.2196/formative.9995)

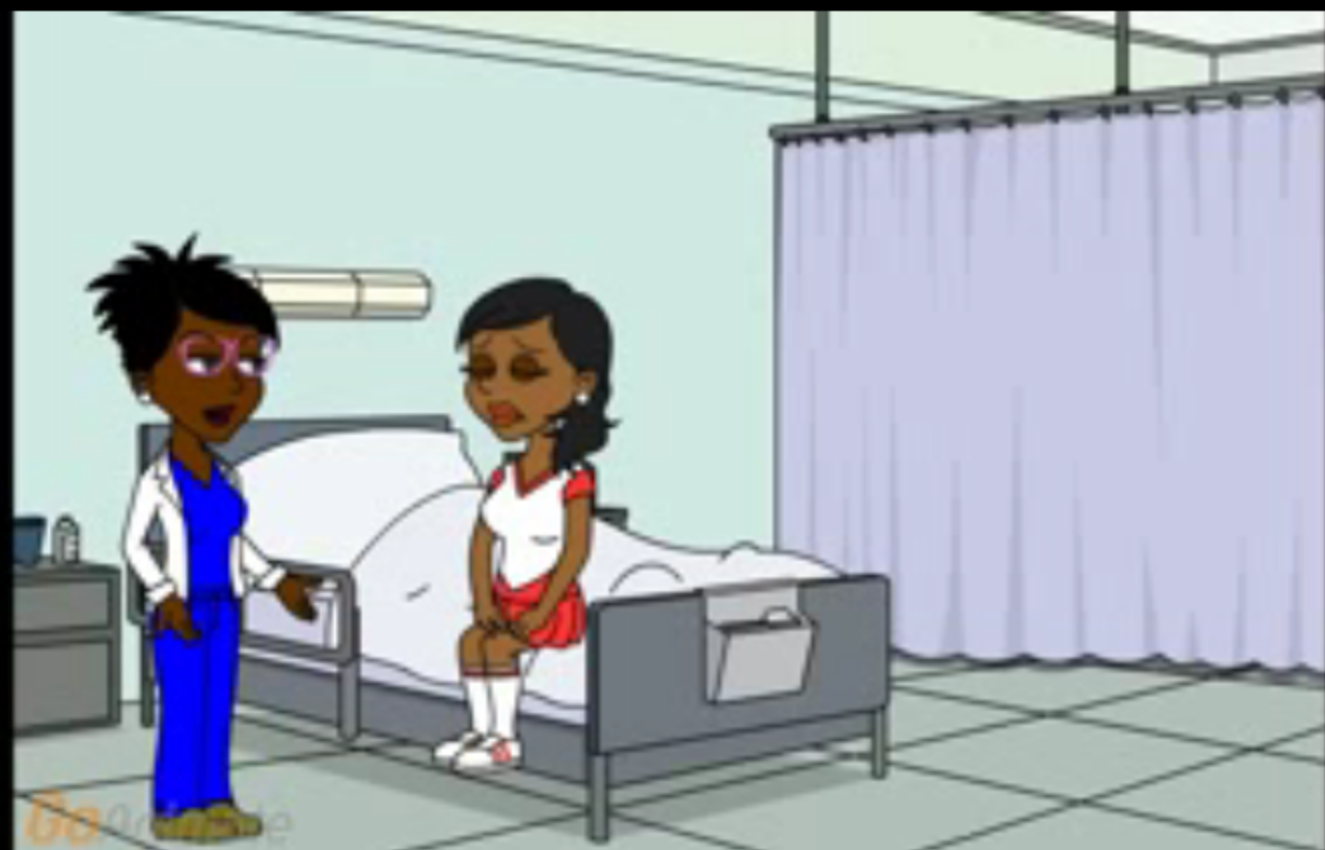

Supplement: Multimedia Appendix 1 [file formative_v3i2e9995_app1.pdf]

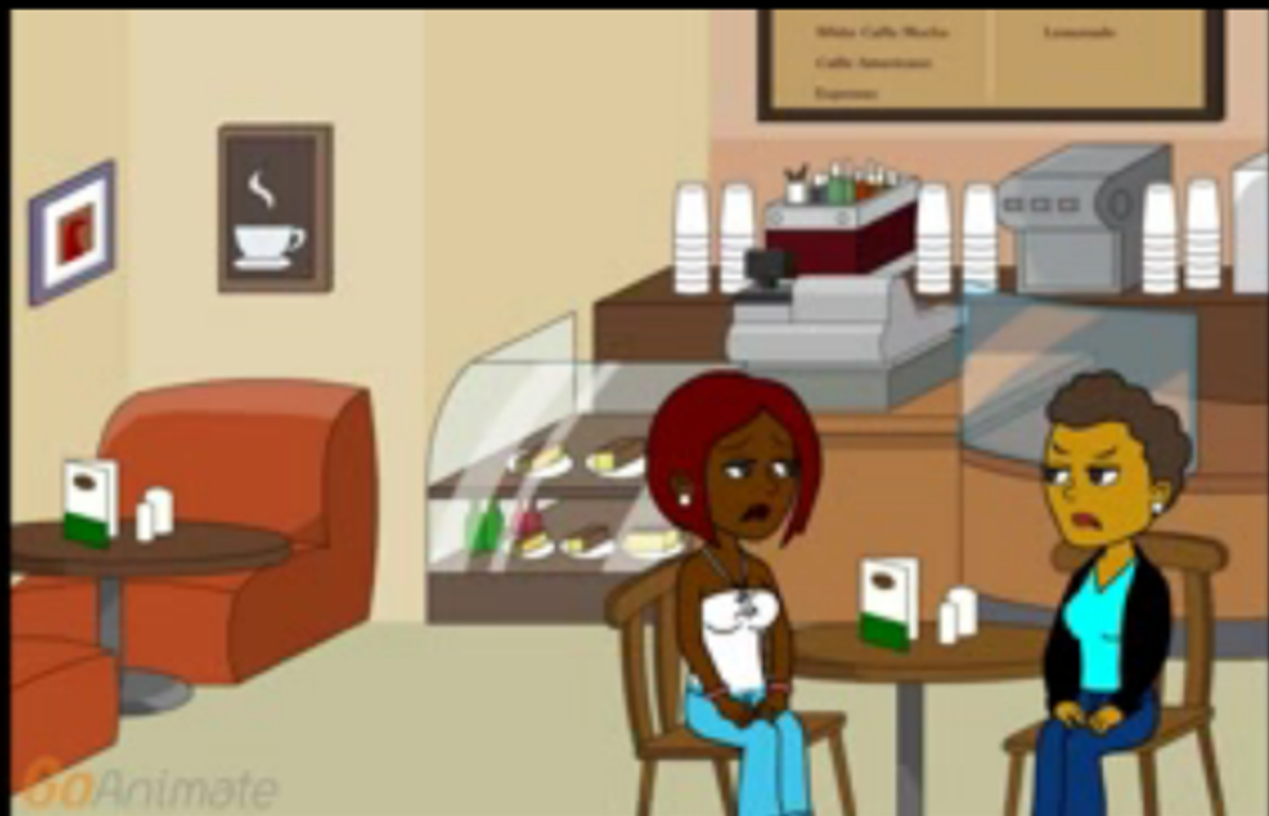

Supplement: Multimedia Appendix 2 [file formative_v3i2e9995_app2.pdf]
